# Satellite-based time-series of sea-surface temperature since 1980 for climate applications

**DOI:** 10.1038/s41597-024-03147-w

**Published:** 2024-03-29

**Authors:** Owen Embury, Christopher J. Merchant, Simon A. Good, Nick A. Rayner, Jacob L. Høyer, Chris Atkinson, Thomas Block, Emy Alerskans, Kevin J. Pearson, Mark Worsfold, Niall McCarroll, Craig Donlon

**Affiliations:** 1 Department of Meteorology, University of Reading, Reading, UK; 2 National Centre for Earth Observation, University of Reading, Reading, UK; 3 Met Office, Exeter, UK; 4 Danish Meteorological Institute, Copenhagen Ø, Denmark; 5 Brockmann Consult GmbH, Hamburg, Germany; 6 Swansea University, Swansea, UK; 7 European Space Agency, Noordwijk, Netherlands

**Keywords:** Climate sciences, Physical oceanography

## Abstract

A 42-year climate data record of global sea surface temperature (SST) covering 1980 to 2021 has been produced from satellite observations, with a high degree of independence from *in situ* measurements. Observations from twenty infrared and two microwave radiometers are used, and are adjusted for their differing times of day of measurement to avoid aliasing and ensure observational stability. A total of 1.5 × 10^13^ locations are processed, yielding 1.4 × 10^12^ SST observations deemed to be suitable for climate applications. The corresponding observation density varies from less than 1 km^−2^ yr^−1^ in 1980 to over 100 km^−2^ yr^−1^ after 2007. Data are provided at their native resolution, averaged on a global 0.05° latitude-longitude grid (single-sensor with gaps), and as a daily, merged, gap-free, SST analysis at 0.05°. The data include the satellite-based SSTs, the corresponding time-and-depth standardised estimates, their standard uncertainty and quality flags. Accuracy, spatial coverage and length of record are all improved relative to a previous version, and the timeseries is routinely extended in time using consistent methods.

## Background & Summary

Sea surface temperature (SST) is one of the 55 Essential Climate Variables (ECVs) as defined by the Global Climate Observing System (GCOS)^1^. It is a key parameter within the Earth’s climate system as most of the climate’s effective thermal inertia is stored in the upper ocean^2^ and SST is the main factor determining the atmospheric response to the ocean^3^. Uses of SST data include quantification of climate change and variability, evaluation of climate and ocean models, input to numerical weather prediction and forecasting systems, and applications in oceanography, maritime safety and operations, fisheries, tourism, and transport^4^. Usable *in situ* measurements of SST date back to the 19^th^ century^5^. These were initially made from ships using manual methods. Automated measurements from buoys became increasingly important during the satellite period. Since the 1980s, satellite observations of top-of-atmosphere radiances have enabled SSTs to be estimated from space, providing more complete spatio-temporal coverage than the *in situ* networks.

The European Space Agency (ESA) Climate Change Initiative (CCI)^6^ is generating satellite-based climate data records (CDRs) that aim to answer the GCOS requirements^7^. This paper presents the third version of the SST CDR generated within the CCI. This version spans 42 years from 1980–2021 inclusive, with an ongoing extension from 2022 onwards provided as an Interim CDR (ICDR) – funded by the Copernicus Climate Change Service (C3S; https://climate.copernicus.eu/) during 2022, with 2023 onwards funded by the UK Earth Observation Climate Information Service (EOCIS) and UK Marine and Climate Advisory Service (UKMCAS). The SST CCI CDR v3.0 was produced using thermal infrared (TIR) and passive microwave (MW) radiances from 22 different satellite platforms. The input radiances were collected by four series of sensors: 15 Advanced Very High Resolution Radiometers (AVHRRs), three Along-Track Scanning Radiometers (ATSRs), two Sea and Land Surface Temperature Radiometers (SLSTRs), and two Advanced Microwave Scanning Radiometers (AMSRs). The spatial footprint of the TIR observations varies from 1 × 1 km (ATSR/SLSTR) to approximately 15 × 1.9 km (AVHRR Global Area Coverage (GAC) assuming satellite zenith of 60 degrees and altitude of 850 km), with valid SSTs produced for cloud-free and ice-free views of the ocean. The MW sensors have a lower spatial resolution, ~50 km, and are able to obtain valid SST measurements through non-precipitating cloud; however, retrievals are not possible in the presence of rain or close to land or ice surfaces. All SSTs include a Quality Level (QL) estimate in the range 0–5, with QLs of 4 and 5 being suitable for climate applications. Furthermore, a datum-specific estimate of the associated uncertainty^8,9^ is provided for all SST values, broken down into components representing different length scales of correlation of SST errors (random, locally correlated, and systematic).

All data are generated following the Group for High Resolution Sea Surface Temperature (GHRSST) Data Specification (GDS)^10^. Data are provided at four product levels: Level-2 pre-processed (L2P): single-sensor data at their native resolution (orbit view); Level-3 uncollated (L3U): single-sensor files remapped to a 0.05° latitude-longitude grid; Level-3 collated (L3C): single-sensor data collated to fixed 24-hour periods (day-time and night-time data are collated separately); and Level-4 analysis (L4): combines data from all sensors using an analysis to produce a daily gap-filled product.

Inter-comparisons and combination of satellite and *in situ* measurements of SST are complicated by the fact that they are measuring different quantities^11^. Satellite-based retrievals are sensitive to the thermally emitted radiance over a large area, but very shallow depth dependent on the wavelength used for observations. In the case of infrared sensors the sensitivity is to the temperature of the top few micrometres of the ocean skin layer^12,13^, and for microwave sensors it is about a millimetre in the sub-skin layer^14^. Meanwhile, *in situ* measurements are typically point measurements at depths from ~10 cm to ~10 m. The temperature difference between the skin and depth SST is typically a few tenths of kelvin, but can be larger particularly in cases of sustained low windspeed or daytime warming of the upper ocean^12–14^. While some satellite SST products average over skin-depth differences via empirical regression, CCI SSTs are physics based^15,16^. Therefore, an estimate of the skin-to- depth (or subskin-to-depth) SST difference is required in order to use the satellite-based CCI SSTs with the historical *in situ* record. Here, we model these differences relative to a depth of 20 cm, corresponding to the nominal depth of drifting buoys^17^ and the approximate depth of historical bucket SST measurements^18^

**Fig. 1:**
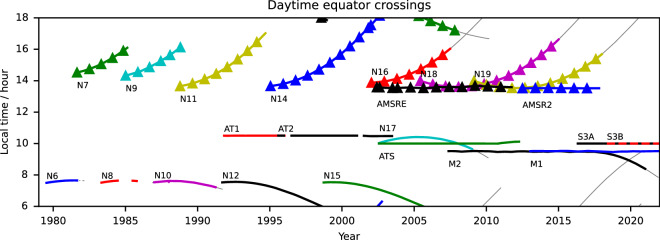
Satellite Equator-crossing times for platforms carrying contributing sensors. Solid lines indicate southward crossings; lines with triangles indicate northward crossings; thin grey lines indicate periods when the satellite is operational, but the sensor data are not used in this work. “N” = NOAA platform carrying an AVHRR. “AT” = ATSR. “M’ = MetOp platform carrying AVHRR processed at full resolution. “S3” = Sentinel 3 platform carrying an SLSTR.

The time of SST measurement is also important due to the diurnal cycle in surface temperature. The diurnal cycle in SST has been empirically characterised from sub-daily drifting buoys^19,20^ and from satellites^21^. The SST undergoes a diurnal warming and cooling cycle with a peak-to-peak range of typically 0.0 to 0.5 K. Under low-wind and high-insolation conditions, the diurnal warming can exceed 5 K^22^. The satellites used to produce the CDR are in a range of orbits with different, often drifting, local overpass times (Fig. 1) which must be accounted for in order to avoid aliasing the diurnal cycle into the long term record^19^. The SST CCI products include an adjustment to the nearest 10:30 or 22:30 local mean solar time, which provides a good approximation to the daily mean SST^20^. The CCI dataset is the only SST CDR to minimise the aliasing of time of day into the long-term climate trend in this way.

**Fig. 2:**
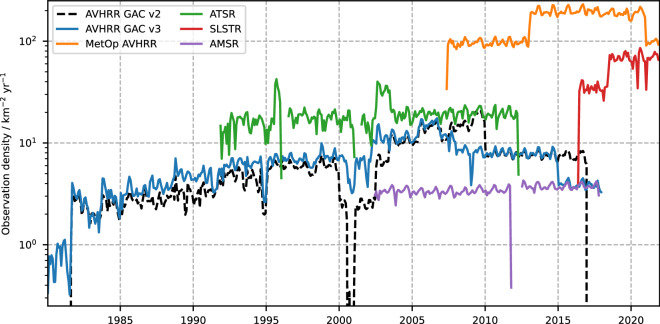
Number of good quality (quality level 4 or 5) SST observations per unit area of ocean (“observation density”) per sensor-type over time. The new version 3 dataset adds AMSR, SLSTR, and full-resolution MetOp AVHRR sensors to the climate data record (CDR). Version 3 also processes more AVHRR data to improve coverage in the 1980s. Note – AVHRR GAC v2 appears to have more observations at times from 2006 onwards as MetOp AVHRR was included in AVHRR GAC v2.

The complete dataset contains approximately 1.4 × 10^12^ good-quality pixels, up from 2.7 × 10^11^ in the previous version. Figure 2 shows the observation density of the Level 2 SST CCI products contributing to the CDR. The addition of the full-resolution MetOp AVHRR and SLSTR sensors in recent decades has significantly increased the coverage at the recent end of the record with over 100 SST estimates km^−2^ yr^−1^. With the more limited satellite coverage in the 1980s the SST CCI dataset becomes more sparse, with only ~5 km^−2^ yr^−1^ in 1990 decreasing to less than 1 km^−2^ yr^−1^ in 1980 when only one AVHRR sensor was operating.

Compared to the previous version 2.1 CDR^23^ the key changes in version 3 (CDR and ICDR) are as follows. The time series is longer, covering 1980 to recent time (compared to Sept 1981 to 2016 for v2.1). An improved representation of radiative effects of tropospheric aerosol is used for cloud detection and SST retrieval, which has greatly reduced the previous few-tenths cold biases associated with desert-dust aerosol. Bias aware optimal estimation^24,25^ is now used to bias-correct radiances prior to single-view AVHRR SST retrieval, which reduces systematic instrumental biases. This in part has also facilitated improved spatio-temporal coverage during the 1980s through the addition of AVHRR/1 sensors and improved AVHRR processing. From 2016 onwards, the dual-view SLSTR data are included. MetOp AVHRR data from 2007 onwards are now processed at full resolution (CDR v2 used lower resolution GAC data for all AVHRRs); this reduces cloud-screening related errors and improves overall coverage. Lastly, AMSR SSTs have been included.

## Methods

**Fig. 3:**
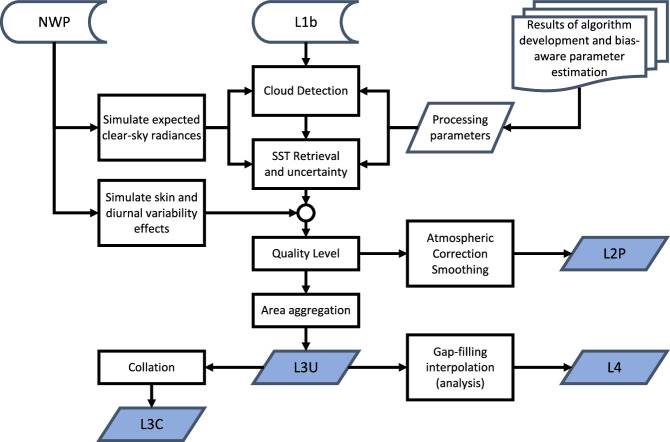
IR Production flowchart. Input data streams are the Level 1b (L1b) satellite imagery (calibrated and geo-located radiances or brightness temperatures), and atmospheric reanalysis (referred to here to as Numerical Weather Prediction – NWP) data. Outputs are SSTs and uncertainties at the same pixel resolution as the input imagery (L2P); averaged onto a 0.05 latitude-longitude grid (L3U, one file per input L1b); collated to single-sensor daily files (L3C); and a multi-sensor gap-filled daily analysis (L4).

**Fig. 4:**
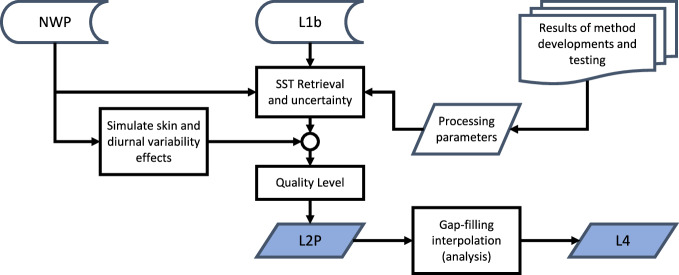
MW production flowchart. See Fig. 3 for explanation of inputs and outputs.

Figure 3 presents an overview of the major processing steps and data flows from Level 1 radiances to the various SST products for infrared sensors, while Fig. 4 shows the equivalent for microwave products. Physically based methods are used wherever possible to minimise the dependence on *in situ* measurements. For infrared sensors this includes both Bayesian methods of cloud screening^26,27^ and SST retrieval^15,28^ which both rely on radiative transfer modelling to simulate clear-sky radiances^16,29^. Microwave retrievals^30^ of SST are not affected by the presence of cloud, though the current retrieval is more directly tied to *in situ* measurements than the infrared. For all sensors, physical modelling of the diurnal cycle is used to produce time and depth-adjusted SSTs in order to avoid aliasing the daily SST cycle into the long-term trends^31,32^, and all retrievals include an associated estimate of uncertainty which is propagated through to the higher level products^33,34^. Finally, SSTs from all sensors are combined to give a gap-free daily SST analysis^35,36^. The data and methods are described in more detail in the next subsections.

### Input satellite data

The 22 satellite datasets used to build the CDR are listed in Table 1, with further details for the various sensor types given in Table 2. The three ATSR-series sensors (ATSR-1^37^, ATSR-2^38^, and AATSR^39^) and two SLSTRs (SLSTR-A^40^, and SLSTR-B^41^) are two-point calibrated, dual-view instruments designed to produce long-term climate quality measurements for SST estimation. For the ATSR-series we use the version 3/2.1 level 1b archive (http://data.ceda.ac.uk/neodc/aatsr_multimission/), while SLSTR is the latest reprocessing as of 2022 (https://data.ceda.ac.uk/neodc/sentinel3a/data/SLSTRhttps://data.ceda.ac.uk/neodc/sentinel3b/data/SLSTR). In the case of SLSTR, the Vis/NIR channels have a higher resolution (500 m) than the thermal infrared (1 km) channels; in combination with the conical scanning geometry this can result in an up to 1 pixel misalignment between the two in the regular image grid^42^. This is addressed by remapping the higher resolution data to the 1 km infrared pixel locations using averaging over five nearest neighbours^43^.

**Table 1:** Summary of characteristics of satellite level-1 data used.

Sensor	Type	Data Start	Data Stop	Daytime Overpass Time
AVHRR-6	AVHRR/1	1979-07-13	1981-08-31	AM
AVHRR-7	AVHRR/2	1981-08-24	1985-02-18	PM
AVHRR-8	AVHRR/1	1983-05-04	1985-10-14	AM
AVHRR-9	AVHRR/2	1985-01-04	1988-11-07	PM
AVHRR-10	AVHRR/1	1987-01-01	1991-03-31	AM
AVHRR-11	AVHRR/2	1988-10-12	1994-09-13	PM
AVHRR-12	AVHRR/2	1991-09-16	1998-12-14	AM
AVHRR-14	AVHRR/2	1995-01-19	2002-10-07	PM
AVHRR-15	AVHRR/3	1998-09-24	2007-12-31	AM
AVHRR-16	AVHRR/3	2002-01-14	2007-06-30	PM
AVHRR-17	AVHRR/3	2002-08-01	2008-12-31	10.00 h
AVHRR-18	AVHRR/3	2005-06-05	2014-12-31	PM
AVHRR-19	AVHRR/3	2009-02-22	2017-12-31	PM
AVHRR MetOp-A	AVHRR/3	2007-05-21	2020-12-31	09.30 h
AVHRR MetOp-B	AVHRR/3	2013-01-15	2021-12-31	09.30 h
AMSRE	AMSR	2002-06-01	2011-10-04	13.30 h
AMSR2	AMSR	2012-07-02	2017-10-26	13.30 h
ATSR-1	ATSR1	1991-11-01	1996-01-09	10.30 h
ATSR-2	ATSR2	1995-08-01	2003-06-22	10.30 h
AATSR	AATSR	2002-07-24	2012-04-08	10.00 h
SLSTR-A	SLSTR	2016-05-01	2021-12-31	10.00 h
SLSTR-B	SLSTR	2018-05-30	2021-12-31	10.00 h

Data start and stop indicates the range used to generate the climate data record. The local time of observation varies significantly for the NOAA AVHRR sensors as illustrated in Fig. 1.

**Table 2:** Summary characteristics of the different sensor types used.

Sensor Type	Highest resolution / km	Swath width / km	Zenith angle	0.6 mm	0.8 mm	1.6 mm	3.7 mm	11 mm	12 mm
AVHRR/1	4.4 × 1.1	2900	0-68	Y	Y		Y	Y	
AVHRR/2	4.4 × 1.1	2900	0-68	Y	Y		Y	Y	Y
AVHRR/3	4.4 × 1.1	2900	0-68	Y	Y	N^a^	Y	Y	Y
MetOp AVHRR	1.1	2900	0-68	Y	Y	N^b^	Y	Y	Y
ATSR1	1	512	0-22, 55			Y	Y^c^	Y	Y
ATSR2	1	512	0-22, 55	N^d^	N^d^	Y	Y	Y	Y
AATSR	1	512	0-22, 55	N^e^	N^e^	Y	Y	Y	Y
SLSTR	1	750	0-30, 55	Y	Y	Y	Y	Y	Y
AMSR	~50	1450	55	N/A – microwave instrument					

Resolution for AVHRRs is given for nadir viewing conditions. For AMSR the resolution varies with channel frequency and is assumed to be ~50 km here; representative of the resolution of SST products. Y indicates the presence and use of a channel on an instrument; N indicates the channel is present, but not used. a: AVHRR/3 added a 1.6 micron channel which could be transmitted during the day instead of the 3.7 micron, but this mode of operation was rarely used. b: MetOp-AVHRRs always provide 1.6 micron data during the day, and 3.7 micron during the night. c: The 3.7 micron channel on ATSR-1 failed early in the mission, but is used where present. d: These channels on ATSR-2 had restricted data availability over oceans so were not used. e: Channels were available on AATSR, but not used.

The 15 AVHRRs are multipurpose imaging instruments which have used three designs over the years, adding further spectral channels and improved sun shielding. They have been used onboard the National Oceanic and Atmospheric Administration (NOAA) Polar Operational Environmental Satellites (POES) and, since 2007, the EUMETSAT Polar System (EPS) MetOp satellites. The AVHRR instrument has a ground resolution of ~1.1 km at nadir; however, hardware limitations when the instruments were originally designed meant it was not possible to record a full orbit of data onboard for transmission to the ground stations. Therefore, the data are processed onboard to produce lower resolution Global Area Coverage (GAC) data. The averaging process for GAC pixels uses the mean of four pixels out of every five, every third line. The newer MetOp satellites do not have this limitation and record full orbit data at native resolution, hence the MetOp AVHRRs are listed separately in Table 2. We obtained the full archives of AVHRR L1b data from the NOAA CLASS archive and combined these with additional orbits obtained from University of Miami (NOAA 6, 7, 9, 11, 15, and 16) and all are now available from the UK Centre for Environmental Data Analysis (CEDA)^44^.

The two passive microwave sensors, AMSR-E onboard EOS-Aqua and AMSR-2 onboard GCOM-W1, can retrieve SST through clouds, allowing measurements in all conditions except rain and within 100 km of land or sea ice. Ground resolution and SST accuracy are poorer than for the infrared sensors. The AMSR instruments use a conical scanning pattern with zenith angle of 55°, which results in an elliptical footprint aligned along the view direction. The size of the AMSR footprint varies with the channel frequency, with the 6.9 GHz channel having sizes of 43 × 75 km and 35 × 62 km for AMSR-E and AMSR2 respectively, and SST products are assumed to have a resolution of ~50 km. AMSR-E L2A Version 3 data were obtained from the NASA NSIDC^45^, and AMSR-2^46^ L1R Version 2 data from JAXA (https://gportal.jaxa.jp). We were able to download and process AMSR2 to October 2017 within available resources; however, this will be extended in future work.

### Auxiliary data

Numerical Weather Prediction (NWP) data are used to drive radiative-transfer modelling of Bayesian prior clear-sky radiances which are required for the cloud detection and some retrieval methods. We use a combination of the European Centre for Medium-range Weather Forecasting (ECMWF) Re-Analysis Interim^47^ (ERA-Interim) and ECMWF Re-Analysis 5^48^ (ERA-5) datasets. The ERA-interim dataset is used when processing NOAA AVHRR and ATSR sensors, while ERA-5 is used for the more recent MetOp AVHRR and SLSTR sensors. It was necessary to combine the two versions of the reanalysis dataset as the full ERA-5 dataset was not available at CEDA during the initial algorithm development work, and ERA-interim ends August 2019. Sensitivity of the results to the NWP source is negligible, by design.

In addition to the atmospheric fields included with the ECMWF NWP, we also require a prior estimate of the surface temperature. While the NWP datasets do include the input SST field used as the lower boundary condition for the atmospheric model, this “NWP SST” is not ideal for our use as the source of the reanalysis SST changes over time with different spatial and temporal resolutions. In order to maximise the consistency in the CDR reprocessing we use the gap-filled SST CCI analysis v2.1^23^ along with a correction for known dust and calibration biases^49^. Importantly, the process used in gap-filling largely decorrelates SST errors in this prior with errors in the TIR imagery even when the same satellite observations are used in both versions, so the assumption of prior independence in optimal estimation is not violated. A climatology of the dust-corrected prior SST is available as the SST CCI Climatology v2.2^50^ and is used to calculate the SST anomalies and within the Level 4 Analysis system.

Tropospheric aerosol data are taken from the Copernicus Atmosphere Monitoring Service (CAMS) reanalysis^51^ and CAMS aerosol climatology^52^. We use all the aerosol components provided in the climatology, although the mineral dust components are the most relevant as they can cause impacts of more than 1 K in the infrared observations, while other aerosol components’ impact is typically 0.1 K or less.

There are three major historical volcanic eruptions (El Chichón^53^, Mount Pinatubo and Mount Hudson^54^) which affect the current dataset by increasing stratospheric sulphate aerosol concentrations with significant impacts on the infrared observations used for SST retrieval^55^. Our estimate of stratospheric aerosol is derived from High-Resolution Infrared Radiation Sounders, using an adaption of a published method^56^. This is the same stratospheric aerosol dataset as was used for the previous CDR^23^.

Sea-ice concentration data from the EUMETSAT Ocean and Sea Ice Satellite Application Facility (OSI-SAF) are used within the Level 4 Analysis system. Two datasets are needed to cover the period processed: OSI-450^57^ to end-2015, and OSI-430-b^58^ for 2016 onwards. These are a CDR and ICDR pair derived from SMMR/SSMI/SSMIS passive microwave observations.

A global distance-to-land dataset^59,60^ derived from ESA Land Cover (LC) CCI data^61^ is used to determine if a given satellite pixel is entirely over water, or overlapping land from its centre location and view angle. The Caspian Sea (excluding the Garabogazköl Basin) is included as ocean; however, lakes are not processed. The same data^60^ is used to produce a fixed land sea mask at 0.05°, with ice shelfs^62,63^ excluded, for use in the Level 4 Analysis system.

Pre-calculated look-up tables (LUTs) are used by the infrared cloud detection scheme (see ‘Cloud Detection’ below) and SST retrieval schemes and are available^64^.

### Radiance Harmonisation

Harmonisation of the infrared radiances (or brightness temperatures) is used for the ATSR and AVHRR-series instruments. This process reconciles the radiometric differences between sensors with the expected differences due to the measured differences in their spectral response functions^65,66^. The radiance-level harmonisation for ATSR and AVHRRs used here is the same as the version 2 CDR^23^. The SLSTR-series of instruments do not overlap with the ATSR-series so no direct harmonisation is possible. However, an investigation using MetOp-A AVHRR to bridge the gap between AATSR and SLSTR found the relative calibration of the two sensors to be compatible^24^.

For the AVHRR instruments, the radiance harmonisation was done as a metrological analysis of the calibration and provides an updated set of AVHRR calibration coefficients used for the counts to radiance conversion along with the estimated instrument uncertainties. While the radiance harmonisation does reduce both inter- and intra-AVHRR retrieval biases, it was still necessary to include a post-hoc adjustment of the AVHRR SSTs in CDRv2.1. For this new version, we instead address the residual instrumental biases as part of the SST retrieval, which is preferable. Corrections are obtained using bias-aware optimal estimation^24,25^, in which an ensemble of satellite-to-reference matches enables inference of instrumental bias relative to radiative transfer, prior bias (in the water vapour profile from NWP), and the error covariances for the measurements and prior.

The NOAA AVHRR instruments were harmonised against *in situ* observations. While *in situ* references are not used to derive empirical SST retrieval parameters nor in the generation of gap-filled products, the use of *in situ* references to adjust NOAA AVHRR brightness temperatures does introduce an element of dependence between some CCI SSTs and the *in situ* SST record in this version.

The MetOp AVHRR instruments were harmonised against AATSR and SLSTR observations^24^. ATSRs and SLSTR act as satellite references, which, combined with physics-based retrieval, gives full independence from *in situ* measurements for the dual-view instruments and MetOP AVHRR.

### Radiative transfer modelling

Both the cloud detection and SST retrieval are physically based methods using a radiative transfer or forward model to estimate the expected clear-sky radiances. Two radiative transfer models are used: a line-by-line model used in the generation of SST retrieval coefficients for dual-view sensors; and a fast parameterised model used for cloud detection and single-view SST retrieval.

The design of the ATSR^67^ and SLSTR^68^ sensors with dual-view scanning geometry and two on-board blackbody calibration sources allow a highly-accurate, aerosol-robust, coefficient-based retrieval of SST^28^. With a coefficient-based scheme the radiative transfer simulations can be performed “offline” so we may use the most accurate model available. Therefore, we use a line-by-line layer-by-layer model to calculate top-of-atmosphere spectral radiances based on a published method^16^. The radiative transfer model is LBLRTM^69^ v12.2 with AER^70,71^ v3.2 spectroscopic data. Channel integrated radiances are calculated by convolving the spectral radiances with the instrument spectral response functions. The trace gas concentrations for CO_2_^72^, CH_4_^73^, N_2_O^74^, CFC-11^75^, and CFC-12^76^ have been updated to cover 1991 to 2021 to ensure that secular trends in gas concentrations do not result in trend artefacts in the SST retrieval.

The cloud detection and optimal-estimation (used for single-view AVHRR sensors) SST retrieval methods require that the radiative transfer simulations are run for each satellite observation to be processed and must calculate the top-of-atmosphere radiances and their partial derivatives with respect to changes in the input state. Due to the large data volumes, computational speed prevents line-by-line calculation, and so a parameterised, fast model has been used, specifically, RTTOV version 12.3 software^29^ with the “lblrtm_v12.8/aer_v_3.6” coefficients released in October 2020.

### Cloud detection (TIR sensors)

Clouds are largely opaque at infrared wavelengths meaning it is not possible for infrared sensors to retrieve SSTs from cloud-filled pixels, while partial presence of cloud can introduce SST errors (because clear-sky retrieval assumptions are violated). Therefore, it is necessary to apply a cloud detection process to satellite imagery to minimise cloud-related errors in the final SST products. Clouds are typically brighter (at visible wavelengths), colder (at infrared wavelengths) and more spatially variable than the underlying sea surface, which provides the basis of cloud detection. Partial or thin cloud cover is harder to detect as the magnitudes of the cloud effects are smaller. The approach used here is to use the radiative transfer model to simulate the radiances expected under clear-sky conditions. The observations are then compared against the clear-sky simulations and a distribution of cloudy-affected radiances to estimate the probability that a given pixel is clear using Bayes’ theorem^26,27,77,78^. The probability, $P\left(c| {\boldsymbol{y}},{{\boldsymbol{x}}}_{a}\right)$, that a pixel is clear-sky given the satellite observations, ***y***, and the prior state, ***x***_*a*_, may be written:

$$P(c| {\boldsymbol{y}},{{\boldsymbol{x}}}_{a})=\frac{P({\boldsymbol{y}}| {{\boldsymbol{x}}}_{a},c)P({{\boldsymbol{x}}}_{a}| c)P(c)}{P({\boldsymbol{y}}| {{\boldsymbol{x}}}_{a})P({{\boldsymbol{x}}}_{a})}$$

Where: ***c*** indicates the required condition (clear-sky, ice-free ocean); the observation vector ***y*** comprises the brightness temperatures (BTs) for thermal channels, reflectances for visible / near infrared channels, and the local standard deviation of BT over the surrounding 3 × 3 pixels; the prior state ***x***_*a*_ includes the atmospheric and surface state from ECMWF NWP data, tropospheric aerosol CAMS, and stratospheric aerosol as described above. The equation is simplified by assuming $P\left({{\boldsymbol{x}}}_{a}| c\right)=P\left({{\boldsymbol{x}}}_{a}\right)$ as the NWP data with length scales ~10 to ~100 km cannot resolve cloud structures at the pixel scales ~1 to ~10 km. The $P\left({\boldsymbol{y}}| {{\boldsymbol{x}}}_{a}\right)$ term can be expressed as the sum over clear-sky *(c*) and not-clear ($\bar{c}$) states to give:

$$P(c|{\boldsymbol{y}},{{\boldsymbol{x}}}_{a})=\frac{P({\boldsymbol{y}}|{{\boldsymbol{x}}}_{a},c)P(c)}{P({\boldsymbol{y}}|{{\boldsymbol{x}}}_{a},c)P(c)+P({\boldsymbol{y}}|{{\boldsymbol{x}}}_{a},\bar{c})P(\bar{c})}$$

The terms on the right-hand side of the equation are calculated as follows. $P({\boldsymbol{y}}| {{\boldsymbol{x}}}_{a},c)$ is the probability density function (PDF) of the observations given the prior state and assuming clear-sky, which, assuming Gaussian errors in the background and observations, can be calculated from the radiative transfer simulations following^27,79,80^:

$$P({\boldsymbol{y}}|{{\boldsymbol{x}}}_{a},c)=\frac{\exp (-0.5{({\boldsymbol{y}}-F({{\boldsymbol{x}}}_{a}))}^{T}{({{\bf{K}}{\bf{S}}}_{a}{{\bf{K}}}^{T}+{{\bf{S}}}_{{\rm{\varepsilon }}})}^{-1}({\boldsymbol{y}}-F({{\boldsymbol{x}}}_{a})))}{{(2\pi )}^{\frac{n}{2}}{|{{\bf{K}}{\bf{S}}}_{a}{{\bf{K}}}^{T}+{{\bf{S}}}_{{\rm{\varepsilon }}}|}^{\frac{1}{2}}}$$

Where $F\left({{\boldsymbol{x}}}_{a}\right)$ is the forward model, the matrix **K** contains the forward model tangent-linears (the derivatives of the forward model output with respect to the prior), **S**_a_ is the error covariance matrix for the prior state, and **S**_*ε*_ is the error covariance for the model and observation differences.

The cloudy-sky PDF, $P({\boldsymbol{y}}|{{\boldsymbol{x}}}_{a},\bar{c})$, is taken from pre-generated lookup tables as documented in ATSR^81^ and AVHRR^26^. The prior probability of clear-sky, *P*(*c*), and cloudy-sky, $P\left(\bar{c}\right)=1-P\left(c\right)$, are obtained from the NWP cloud fraction, constrained to the range 0.5 to 0.95, representing the probability of cloud at an arbitrary point within the NWP grid cell.

### Retrieval of skin SST (dual-view sensors)

For the dual-view ATSR and SLSTR sensors the SST retrieval is based on the method developed for the ATSR Reprocessing for Climate project^16,28^ which is a coefficient-based retrieval using the equation:

$$\widehat{x}={a}_{0}+{{\boldsymbol{a}}}^{{\rm{T}}}{\boldsymbol{y}}$$

Where $\widehat{x}$ is the retrieved SST, *a*_0_ is the offset coefficient, ***a*** is a vector of n weights or coefficients to multiply the observed brightness temperatures, ***y***. The coefficients are pre-calculated using a least-squares minimisation technique from the line-by-line radiative transfer simulations. The coefficients are defined for a range of parameters: satellite zenith angle in the nadir-view; satellite zenith angle in the forward (or oblique) view, prior total column water vapour, instrument detector temperature (ATSR-1 only), and year (to account for changes in trace gas concentration). The coefficients are linearly interpolated to the state of the pixel being processed.

The uncertainty in the retrieval is separated into two components: independent (uncorrelated) and systematic (correlated). The uncorrelated uncertainty arises from radiometric noise in the satellite observations, and can be calculated by propagating the instrument noise (expressed as a covariance matrix S_*o*_) through the retrieval^28^ as:

$${u}_{unc}=\sqrt{{{\boldsymbol{a}}}^{{\rm{T}}}{{\rm{S}}}_{o}{\boldsymbol{a}}}$$

The systematic (correlated) uncertainties^82^ arise due to errors in the instrument calibration and characterisation, forward model simulation, and prior state. The dual-view retrieval algorithm is designed to minimise these, and the correlated uncertainty is taken to be given by the fitting error when generating the coefficients. This is tabulated along with the retrieval coefficients and interpolated to the pixel conditions^28,33^.

When deriving the SST retrieval coefficients there is a trade-off between uncorrelated uncertainties, correlated uncertainties, and retrieval sensitivity^83^ (retrieval response to changes in true SST). For climate products, the priority is high retrieval sensitivity and minimised correlated uncertainties, because uncorrelated uncertainties are reduced through averaging in the gridded products and long-term time series. To avoid full resolution SST imagery (L2P) being unduly noisy, however, we calculate SST for the full resolution products using “atmospheric correction smoothing”^84^.

Atmospheric correction smoothing exploits the fact that water vapour in the atmosphere tends to vary on longer length-scales than the pixels. A generalised atmospheric correction (a difference between SST and a weighted top-of-atmosphere brightness temperature) can be written as:

$$\delta =x-{{\boldsymbol{b}}}^{{\rm{T}}}{\boldsymbol{y}}$$

where the elements of the vector ***b*** satisfy ${b}_{i}\ge 0$ for all *i* and sum to one. We set the nadir elements of ***b*** to be inversely proportional to the square of the radiometric noise of the corresponding brightness temperature, and set non-nadir elements to zero. The correction, *δ*, can be smoothed over a local area (we use 5 × 5 pixels) to give an atmospherically smoothed SST of:

$$\widetilde{x}=\left\langle \delta \right\rangle +{{\boldsymbol{b}}}^{{\rm{T}}}{\boldsymbol{y}}$$

which is calculated using its equivalent:

$$\widetilde{x}=\left\langle \widehat{x}\right\rangle +{{\boldsymbol{b}}}^{{\rm{T}}}\left({\boldsymbol{y}}-\left\langle {\boldsymbol{y}}\right\rangle \right)$$

This form emphasises that this is not a simple smoothing of the SST itself. In order to avoid cloud contamination affecting the smoothing process, the averaging over the 5 × 5 box only includes pixels with a quality level greater than or equal to the central pixel.

### Retrieval of skin SST (AVHRR-series)

Observations from the single-view AVHRR sensors have a lower information content than the dual-view sensors, so single-view coefficient-based retrievals are often associated with systematic geographic biases^83^. Therefore, we estimate the SST using an optimal estimation retrieval^15,85^ which explicitly includes prior information. The retrieved state, $\widehat{z}$, is expressed as the prior state, ${z}_{a}$ (typically taken from ERA5 NWP data), plus an increment derived from the satellite observations and radiative transfer simulations.

$$\widehat{{\boldsymbol{z}}}={{\boldsymbol{z}}}_{a}+{\bf{G}}\left({\boldsymbol{y}}-F\left({{\boldsymbol{x}}}_{a}\right)\right)={{\boldsymbol{z}}}_{a}+{\left({{\bf{K}}}^{{\rm{T}}}{{\bf{S}}}_{\varepsilon }^{-1}{\bf{K}}+{{\bf{S}}}_{a}^{-1}\right)}^{-1}{{\bf{K}}}^{{\rm{T}}}{{\bf{S}}}_{\varepsilon }^{-1}\left({\boldsymbol{y}}-F\left({{\boldsymbol{x}}}_{a}\right)\right)$$

Here, ***x***_*a*_ includes the full prior state, $F\left({{\boldsymbol{x}}}_{a}\right)$ is the forward simulation for this prior, the matrix **K** contains the partial derivatives of the simulation with respect to the state variables in ${z}_{a}$, **S**_*a*_ is the error covariance matrix for the prior state, and ${{\bf{S}}}_{\varepsilon }$ is the error covariance for the model and observation differences. The reduced state vectors, $\widehat{z}$ and ${z}_{a}$, comprise the SST and total column water vapour.

The uncorrelated and correlated uncertainty components are calculated as:

$${u}_{unc}=\sqrt{{{\bf{GS}}}_{o}{{\bf{G}}}^{{\rm{T}}}}$$

$${u}_{cor}=\sqrt{{{\bf{GS}}}_{{\boldsymbol{\varepsilon }}}^{{\prime} }{{\bf{G}}}^{{\bf{T}}}+\left({\bf{GK}}-{\bf{I}}\right){{\bf{S}}}_{{\bf{a}}}{\left({\bf{GK}}-{\rm{I}}\right)}^{{\rm{T}}}}$$

where S_*o*_ is the error covariance of the radiometric noise in the satellite observations and ${{\rm{S}}}_{\varepsilon }^{{\prime} }$ is the non-noise component of the model-observation error covariance matrix.

Optimal estimation will give the minimum-error retrieval assuming both the prior state and forward model are unbiased, and the error covariance matrices are well known^85^. In practice these assumptions are inexactly met. The original specification for the AVHRR instruments was an accuracy of 1 K for the AVHRR/1 and /2 design and 0.5 K for the later AVHRR/3 design^86^, as such the calibration accuracy of the AVHRR sensors means the forward model cannot be an unbiased representation of the instrument to the accuracy required («0.1 K), and the error covariance matrices must be estimated^25,87^. Here, we correct for the instrument-model bias and empirically estimate the error covariance matrices via bias-aware optimal estimation^24,25^.

### Retrieval of sub-skin SST (AMSR-series)

A two-step multiple linear regression algorithm^30^ is used for microwave SST retrievals as the relationship between the retrieved variables and BT is less linear than the infrared case. This first estimates the wind speed, and then uses that estimate in the SST retrieval. The first-guess windspeed is given by:

$${{\rm{W}}{\rm{S}}}_{a}={a}_{0}+\mathop{\sum }\limits_{i=1}^{10}({a}_{1i}{t}_{i}+{a}_{2i}{t}_{i}^{2})+{a}_{3}({\theta }_{sat}-55)$$

Where *a*_0_, *a*_1*i*_, *a*_2*i*_, and *a*_3_ are global regression coefficients with index *i* representing the channel number up to 10 (the 89 GHz channel is not used for wind speed estimation). The vector ***t*** contains the observed brightness temperatures as ${t}_{i}={BT}_{i}-150$ for all channels except the 23.6 GHz which is ${t}_{i}={\rm{l}}{\rm{n}}(290-{BT}_{i})$. A second-stage accounts for the non-linearities using localised retrieval coefficients. This uses the same form as the first-stage with a different set of regression coefficients, tabulated as a function of the first-guess wind speed.

The SST retrieval uses a similar two-stage approach with the first guess SST given by:

$${{\rm{S}}{\rm{S}}{\rm{T}}}_{c}={c}_{0}+\mathop{\sum }\limits_{i=1}^{12}({c}_{1i}{t}_{i}+{c}_{2i}{t}_{i}^{2})+{c}_{3}({\theta }_{sat}-55)+{c}_{4}{\rm{W}}{\rm{S}}+\mathop{\sum }\limits_{j=1}^{2}({c}_{5j}\cos (j{\phi }_{rel})+{c}_{6j}\sin (j{\phi }_{rel}))$$

Where the initial SST retrieval coefficients, *c*, are tabulated as a function of latitude and orbit direction. WS is the retrieved windspeed, and ${\phi }_{rel}$ is the relative azimuth between the satellite view direction and the wind direction (taken from NWP). For the second-stage retrieval the coefficients are tabulated as a function of the retrieved windspeed and first-guess SST.

The retrieval uncertainties are given by another set of regression equations of the form:

$$u={e}_{0}+{e}_{1}{{\rm{SST}}}_{d}+{e}_{2}{{\rm{SST}}}_{d}^{2}+{e}_{3}{{\rm{WS}}}_{b}+{e}_{4}{{\rm{WS}}}_{b}^{2}+{e}_{5}{\theta }_{sat}+{e}_{6}{\theta }_{sat}+\mathop{\sum }\limits_{p=1}^{4}\left({e}_{7p}\cos \frac{{\phi }_{lat}}{p}+{e}_{8p}\sin \frac{{\phi }_{lat}}{p}\right)$$

With two sets of regression coefficients, *e*, defined: one to estimate uncorrelated uncertainty, *u*_*unc*_, and one for correlated uncertainty *u*_*cor*_.

### Quality level

A measure of the SST data quality is provided on a scale from 0 (no data) to 5 (best quality data). This follows the international convention for SST products^10^. Quality levels 4 and 5 are suitable for use in climate applications where absolute accuracy of SST is important. Users may find the lower quality level data are useful for some applications e.g. detecting SST front locations which only requires relative, not absolute, accuracy.

For the infrared products (ATSR, AVHRR, SLSTR), quality level represents the confidence held both in the SST and in the associated SST uncertainty estimate^9^. A measurement with high uncertainty is still flagged as high quality provided a reliable retrieval is obtainable with a valid uncertainty estimate. Factors which affect the quality level include: cloud detection (pixels with lower probabilities of being clear-sky are assigned lower quality levels), estimated retrieval sensitivity to the true SST, chi-square goodness of fit of retrieval (for optimal estimation retrievals). Additionally, pixels with satellite zenith greater than 60° are considered “limb” pixels with maximum quality level of 2. Pixels with solar zenith angles between 87.5° and 92.5° are classed as twilight and have a maximum quality level of 3. The complete list of quality level checks and thresholds for infrared instruments is shown in Table 3 – pixels will be assigned the lowest level which matches any of the conditions shown.

**Table 3:** Quality level definitions and thresholds used for infrared sensors.

Level	Meaning	P(clear)	Sensitivity	χ2	Other
0	No data	<0			No data; land pixel
1	Bad data	<0.5	<0.0	>3	SST < 271.15 K; ice detected; NWP missing
2	Worst quality	<0.8	<0.10	>2	Limb (θ_sat_ > 60)
3	Low quality	<0.9	<0.20	>1	Twilight (87.5 < θ_sol_ < 92.5)
4	Acceptable quality				ATSR: Aerosol detected: DDI > 0.2 AVHRR: solar contamination detected
5	Best quality				

Pixels will be assigned to the lowest level which matches a condition, therefore quality level 4 and 5 pixels will have P(clear) > = 0.9; sensitivity >= 0.2; and χ^2^ <= 1. P(clear) is the posterior probability of the pixel being clear. “Sensitivity” is the evaluated retrieval sensitivity to true changes in SST. χ^2^ is the channel-normalised goodness of fit test on the retrieval (optimal estimation SSTs only). θ_sat_ is the satellite zenith angle. θ_sol_ is the solar zenith angle. “DDI” is an index for detecting desert or mineral dust^126^.

For microwave sensors the quality level is mainly driven by the estimated uncertainty through the thresholds shown in Table 4. Rain and radio frequency interference (RFI) are flagged as bad data using an additional set of tests^30^, while retrievals within 200 km of sea-ice or 100 km of land are set to quality level 2.

**Table 4:** Quality level definitions and thresholds used for microwave sensors.

Level	Meaning	Uncertainty	Other
0	No data		No data; land pixel
1	Bad data		SST < 271.15 K; SST > 308.15 K; Rain; RFI
2	Worst quality	*u* ≥ 1.0	Proximity to sea ice; proximity to land
3	Low quality	0.5 < *u* < 1.0	
4	Acceptable quality	0.35 < *u* < 0.5	
5	Best quality	*u* ≤ 0.35	

### Adjustments for depth and time

Satellite estimates of SST are based on the thermal radiance from the ocean: this is dependent on the temperature in the top few microns (skin temperature) for infrared sensors, and the top few millimetres (sub-skin temperature) for microwave sensors. These will differ from the depth SST as measured *in situ* by buoys and ships^14^, or used as the upper layer of an ocean model. Therefore, the products include an adjustment that can be added to the primary observation (skin or sub-skin) to give an estimate of the SST at 20 cm depth (comparable to drifting buoy measurement depth).

Furthermore, the local time of observation will depend on the satellite orbit (Fig. 1 and Table 1), with different sensors observing at different times of day. The changing time of observation will cause aliasing of the diurnal cycle into the final record. So, the products include an adjustment for the observation time to avoid spurious inter-annual trends. The adjustment for time-of-day effects is to estimate the SST at the nearest of 10:30 or 22:30 local mean solar time, which is a good approximation to the daily mean SST^20^. The time and depth adjustments are combined to provide SST at 20 cm and 10:30 h or 22:30 h local time in the products.

The time and depth adjustments are calculated using a diurnal variability model^32,88^ which combines a skin-effect model^89^ with a one-dimensional turbulence closure model^90^. The model is driven by NWP surface fluxes and wind stress. The uncertainty associated with this adjustment is also calculated and included in the total uncertainty estimate for the daily mean SST at 20 cm depth.

### Gridded SST products

Level 3 gridded versions of the infrared data are provided on a 0.05° latitude-longitude grid. (The microwave data are not remapped to Level 3 as the sensor resolution is coarser than the 0.05° grid used here.) There are two stages to generating gridded products. First, level 3 uncollated grids (L3U) are made from the full resolution products (L2P) by averaging only the highest available quality level pixels in each gridded cell (so only pixels of the same quality level will be combined). When propagating the uncertainties from level 2 to level 3, the uncorrelated uncertainty (from random errors) decreases with the familiar “$1/\sqrt{n}$” averaging, while the other components do not as the grid size is much smaller than the correlation length scales. When some pixels have been excluded from the final average (e.g. due to cloud cover or a mix of quality levels in the input), then there is an additional uncertainty due to under-sampling. The sampling uncertainty is parameterised as a function of the fraction of pixels used in the cell and the variability in SST for the observed fraction^34^.

Next, all the L3U SSTs from each individual sensor are collated to daily L3C (gridded daily products). Here the content of a L3C grid cell is the “best” L3U SST value acquired in a given 24-hour period, with day and night observations provided separately. The “best” observation is taken to as the one with the highest quality level; if multiple observations have the same quality level, then the observation with the lowest estimated uncertainty is selected.

### Analysed SST

The SST analyses were generated using a climate configuration of the Operational Sea Surface Temperature and Ice Analysis (OSTIA) system^35,91^. OSTIA uses the NEMOVAR data assimilation scheme^92^ to combine multiple input datasets with a background (first guess) field to generate daily, global, gap-free SST and ice concentration analyses. Here we focus on differences in the climate configuration compared to the near real-time version^91^.

For the climate configuration, the only input observational data to the SST analyses are the SST CCI TIR and MW SSTs adjusted to 10:30/22:30 local time and 20 cm depth and their uncertainty estimates. To reduce the data volume being processed by NEMOVAR in the later part of the record when multiple sensors were operational simultaneously, some of the L3U AVHRR data were combined to make super-collated (L3S) input files prior to processing in the OSTIA system. AVHRR data were merged in the following periods (highest priority sensor listed first): August 2002 to end 2007 – NOAA-18, NOAA-16, NOAA-15, NOAA-14; May 2007 to end-2008 – MetOp-A, NOAA-17; February 2009 to end-2014 – NOAA-19, NOAA-18; May 2018 to end-2019 – MetOp-B, MetOp-A. MW data were thinned to 25 km to account for their lower resolution compared to TIR data. Only data files with file quality level set to the highest value (3) were used except for ATSR2 files between 08/02/2001 and 05/07/2001, which had a lower file quality value due to a gyroscope failure on the satellite. From the accepted files, only SSTs with quality level of 4 or 5 were used with the exception of AVHRR data from NOAA-15, for which only data of quality level 5 were utilised. The SST CCI Analysis does not ingest any *in situ* data and all satellite inputs are SST CCI products, so no additional bias correction is performed within the analysis system.

Each daily SST analysis was formed from data from the analysis day and the day either side of it. The uncertainty values for the days either side of the analysis day were inflated by a third to reduce their influence. As with the near real-time system, the background to the analyses was formed by damped persistence of anomalies from the previous day. However, for the climate configuration the anomalies were derived from the SST CCI Climatology version 2.2^50^ (which includes corrections applied for desert dust biases^49^). Although the SST analysis uncertainty estimates were produced using the same method as the near real-time version^91^, it was identified that some of the auxiliary files used in their calculation in the near real-time system have a blocky appearance which propagates into the uncertainty fields. Therefore, for the SST CCI processing, these files were regenerated by interpolating equivalent data held on a different grid to remove the blocky effect.

As described previously, the sea ice concentration input data used for the SST CCI analyses were from the EUMETSAT OSI-SAF OSI-450^57^ and OSI-430-b^58^ products. Visual inspection of the data was performed and days containing suspect features were excluded. Interpolation was carried out to create files for days with missing data using the method described in the SST CCI Algorithm Theoretical Basis Document v1^93^.

Internally within the OSTIA system, the analyses are generated on the extended ORCA12 tripolar grid with 1/12° nominal resolution^94^. The analyses are then regridded onto the 0.05° grid used for the final products. This allows for some flexibility with the land/sea mask applied to the 0.05° gridded data, with the common SST CCI land/sea mask being used in the climate configuration.

### Calibration spike adjustment (L4)

Comparison of the initial Level 4 SST was against HadSST.4.0.1.0^5^, a gappy dataset of gridded *in situ* data, using a method^49^ developed for the previous CDR where the CCI analysis is averaged to the HadSST spatial resolution before estimating an area-weighted global-mean across observed cells. The spatial-mean monthly differences reveal intermittent excursions of 0.1 to 0.2 K in the satellite data during the 1980s and early 1990s. We attribute these “spikes” mainly to periods of unstable calibration of the early AVHRR sensors. Spikes are more noticeable when the record relies on fewer input sensors (see Fig. 1). The magnitudes of the spikes are markedly reduced compared to the previous version 2.1 CDR, which had monthly excursions of up to 0.6 K^23,49^.

**Fig. 5:**
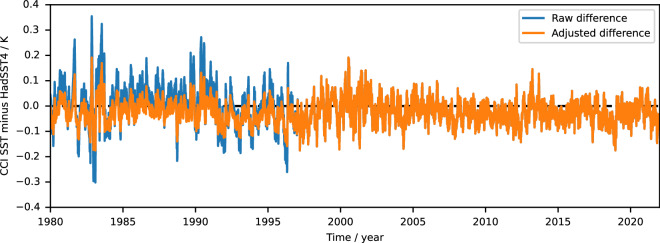
Timeseries of daily global-mean difference of SST CCI analysis minus HadSST4 (K).

To improve the overall homogeneity of the data record at monthly-global scales, we apply a global empirical adjustment to the data up to end-1996, the year of the final major spike in Fig. 5 and corresponding to the gap in ATSR2 data during 1996 evident in Fig. 1. After this point the ATSR2/AATSR, and later SLSTR sensors, provide an independent SST estimate and the empirical adjustment is not necessary. The SST CCI analysis minus HadSST4 differences are distributed near-normally, with standard deviations 0.083 K before 1997 and 0.048 K afterwards. Matching the quantiles of the two distributions results in a near-linear adjustment function (shown in the Algorithm Theoretical Basis Document^95^) that can be used to homogenize the difference distribution of the earlier data to the later data^49,95^. The impact of the adjustment is shown in Fig. 5.

## Data Records

All data records^96–107^ are archived at CEDA: https://archive.ceda.ac.uk/ and made available through the ESA Open Data Portal: https://climate.esa.int/en/data/, under the Creative Commons Attribution 4.0 International license (CC-BY 4.0; https://creativecommons.org/licenses/by/4.0/).

ESA SST CCI products are provided at four different processing levels as defined by the Group for High Resolution Sea Surface Temperature (GHRSST) Data Specification (GDS)^10^. These are shown in the documentation^108^ and previous article^23^ and listed below:

- **Level 2 pre-processed (L2P)**: SST retrievals derived from the Level 1 observations on the Level 1 grid (typically the satellite swath projection), with ancillary data and metadata added following the GDS^10^.
- **Level 3 uncollated (L3U)**: Level 2 data remapped to a regular latitude/longitude grid without combining observations from multiple input files.
- **Level 3 collated (L3C)**: SST observations from a single instrument combined into a space-time grid. For ESA SST CCI products this means collation to daily files.
- **Level 4 analysis (L4)**: SST observations from multiple instruments combined using an analysis system to produce a gridded gap-free product.

The climate data record comprises 14 datasets: 13 for individual sensor families and product levels (L2P to L3C), and one spatially complete analysis product (L4). All files are in netCDF-4 classic format^109^ following: Climate-Forecast (CF) metadata conventions^110^, GHRSST Data Specifications^10^, and ESA CCI Data Standards^111^. The dataset title, full name, description, and other details are given in Tables 5 to 9. The key variables present in the files are listed in Table 10 (Level 2 and 3 products) and Table 11 (Level 4 products).

**Table 5:** Data record information for SST CCI ATSR products.

Title	ESA SST CCI ATSR L2P v2.1^96^	ESA SST CCI ATSR L3U v2.1^97^	ESA SST CCI ATSR L3C v2.1^98^
**Full name**	European Space Agency Sea Surface Temperature Climate Change Initiative: Along-Track Scanning Radiometer		
	level-2 pre-processed product version 2.1	level-3 uncollated product version 2.1	level-3 collated product version 2.1
**Basic description (quotable when citing data)**	global sea surface temperatures from Along Track Scanning Radiometers,		
	presented on the native geometry of observation, and spanning 1991 to 2012	presented on a 0.05° latitude-longitude grid, and spanning 1991 to 2012	daily collations on a 0.05° latitude-longitude grid, and spanning 1991 to 2012
**Data volume**	2.6 T	270 G	242 G
**Acronym (product)**	SST CCI ATSR	Gridded SST CCI ATSR	Gridded daily SST CCI ATSR
**Acronym (SST)**	CCI ATSR SST	CCI gridded ATSR SST	CCI gridded daily ATSR SST

**Table 6:** Data record information for SST CCI SLSTR products.

Title	ESA SST CCI SLSTR L2P v3.0^99^	ESA SST CCI SLSTR L3U v3.0^100^	ESA SST CCI SLSTR L3C v3.0^101^
**Full name**	European Space Agency Sea Surface Temperature Climate Change Initiative: Sea and Land Surface Temperature Radiometer		
	level-2 pre-processed product version 3.0	level-3 uncollated product version 3.0	level-3 collated product version 3.0
**Basic description (quotable when citing data)**	global sea surface temperatures from Sea and Land Surface Temperature Radiometers,		
	presented on the native geometry of observation, and spanning 2016 to 2021	presented on a 0.05° latitude-longitude grid, and spanning 2016 to 2021	daily collations on a 0.05° latitude-longitude grid, and spanning 2016 to 2021
**Data volume**	5.0 T	915 G	175 G
**Acronym (product)**	SST CCI SLSTR	Gridded SST CCI SLSTR	Gridded daily SST CCI SLSTR
**Acronym (SST)**	CCI SLSTR SST	CCI gridded SLSTR SST	CCI gridded daily SLSTR SST

**Table 7:** Data record information for SST CCI AVHRR products.

Title	ESA SST CCI AVHRR L2P v3.0^102^	ESA SST CCI AVHRR L3U v3.0^103^	ESA SST CCI AVHRR L3C v3.0^104^
**Full name**	European Space Agency Sea Surface Temperature Climate Change Initiative: Advanced Very High Resolution Radiometer		
	level-2 pre-processed product version 3.0	level-3 uncollated product version 3.0	level-3 collated product version 3.0
**Basic description (quotable when citing data)**	global sea surface temperatures from Advanced Very High Resolution Radiometers,		
	presented on the native geometry of observation, and spanning 1980 to 2021	presented on a 0.05° latitude-longitude grid, and spanning 1980 to 2021	daily collations on a 0.05° latitude-longitude grid, and spanning 1980 to 2021
**Data volume**	28 T (23 metop)	5.2 T (1.9 metop)	4.3 T (1.4 metop)
**Acronym (product)**	SST CCI AVHRR	Gridded SST CCI AVHRR	Gridded daily SST CCI AVHRR
**Acronym (SST)**	CCI AVHRR SST	CCI gridded AVHRR SST	CCI gridded daily AVHRR SST

**Table 8:** Data record information for SST CCI AMSR product.

Title	ESA SST CCI AMSR L2P v3.0^105^
**Full name**	European Space Agency Sea Surface Temperature Climate Change Initiative: Advanced Microwave Scanning Radiometer level-2 pre-processed product version 3.0
**Basic description (quotable when citing data)**	global sea surface temperatures from Advanced Microwave Scanning Radiometers, presented on the native geometry of observation, and spanning 2002 to 2017
**Data volume**	391 G
**Acronym (product)**	SST CCI AMSR
**Acronym (SST)**	CCI AMSR SST

**Table 9:** Data record information for SST CCI Analysis product.

Title	ESA SST CCI Analysis v3.0^106^	ESA SST CCI Climatology v3.0^107^
**Full name**	European Space Agency Sea Surface Temperature Climate Change Initiative: Analysis product version 3.0	European Space Agency Sea Surface Temperature Climate Change Initiative: Climatology product version 3.0
**Basic description (quotable when citing data)**	daily-mean sea surface temperatures, presented on global 0.05° latitude-longitude grid, with gaps between available daily observations filled by statistical means, spanning 1980 to 2021	daily climatological mean sea surface temperature on a global 0.05° latitude-longitude grid, derived from the SST CCI analysis data for the period 1991 to 2020 (30 years)
**Data volume**	230 G	20 G
**Acronym (product)**	SST CCI analysis	SST CCI climatology
**Acronym (SST)**	CCI analysis SST	CCI climatology SST

**Table 10:** Key data variables present in Level 2 and 3 files.

Name	Units	Description
sea_surface_temperature	K	SST as measured by the satellite sensor (Retrieved SST). For infrared radiometers this is the skin temperature, corresponding to a depth of approximately 10 µm.
sea_surface_temperature_total_uncertainty	K	Total estimated uncertainty in sea surface temperature variable.
sea_surface_temperature_depth	K	SST adjusted to a standard depth of 20 cm and 10:30 h or 22.30 h local solar time (usable as a daily average estimate).
sea_surface_temperature_depth_total_uncertainty	K	Total estimated uncertainty in SST_0.2m_.
sea_surface_temperature_depth_anomaly	K	Difference between SST_0.2m_ and SST CCI Climatology v2.2^50^.

**Table 11:** Key data variable present in Level 4 Analysis files.

Name	Units	Description
analysed_sst	K	Daily mean estimate of SST at 0.2 m depth (Analysed SST).
analysed_sst_uncertainty	K	Estimated uncertainty in Analysed SST.
sea_ice_fraction	—	Areal fraction of sea ice, from 0 to 1.

**ATSR**. The single-sensor ATSR processing (Table 5) has not changed from the previously published version^23^, so the existing v2.1 datasets were used as input to the CDRv3 analysis.

**SLSTR**. The dual-view SLSTR products are listed in Table 6, these start in June 2016 and continue to the end of the CDR. Unlike other sensors processed here, the input SLSTR data are provided in 3-minute granules resulting in ~365 L2P and L3U files per day.

**AVHRR**. Table 7 lists the AVHRR data products which cover the full period of the CDR, with NOAA AVHRR available from 1980 to end-2017, and MetOp AVHRR from May 2007 to 2021.

**AMSR**. The AMSR product is shown in Table 8. Level 3 products are not produced from AMSR-E and AMSR2.

**Analysis**. The spatially complete Level 4 SST CCI analysis product is shown in Table 9.

**Climatology**. A 30-year (1991–2020) daily climatology of the SST CCI analysis product calculated using a 5-day running mean.

All data are released under the Creative Commons Attribution 4.0 International License (CC-BY 4.0, https://creativecommons.org/licenses/by/4.0).

## Technical Validation

### Validation against *in situ* measurements

The SST products are validated against *in situ* measurements as outlined in the Product Validation Plan^112^ and complete results are presented in the Product Validation and Intercomparison Report (PVIR)^108^. An overview of validation is presented here for the Level 3 and 4 products (as used by the majority of users). The *in situ* SST measurements are extracted from the Met Office Hadley Centre Integrated Ocean Dataset (HadIOD) v1.2.0.0^113^, the reference data are referred to as the SST CCI Independent Reference Data Set (SIRDS), see https://www.metoffice.gov.uk/hadobs/hadiod/sirds.html. Collocations between the satellite and *in situ* products are generated using the Multi-sensor Matchup System (MMS)^114^. Due to the changes in the *in situ* coverage since 1980 it is necessary to vary the matchup criteria used as shown in Table 12. For recent sensors (operating since mid 1990s) we can get sufficient validation data with good global coverage using *in situ* drifting buoys (aka “drifters”).

**Table 12:** Matchup criteria used for different sensors and products.

Product / Sensors	Spatial Criterion	Temporal Criterion	*In situ* types
NOAA-06 to 12	12 km	12 hours	All non-ship observations^a^
NOAA-14 to 19	12 km	4 hours	Drifters
ATSR-1	1 km	4 hours	All non-ship observations^a^
ATSR-2 / AATSR	1 km	4 hours	Drifters
MetOp AVHRR	0.025°	2 hours	Drifters
SLSTR	0.025°	2 hours	Drifters
Analysis (to 1996)	0.025°	12 hours^b^	CTD, Drifter, GTMBA, Moorings, XBT
Analysis (1996+)	0.025°	12 hours	Drifters

Where spatial criterion is specified in kilometres the matchup is initially generated against the pixel-level 1 and 2 data; when criteria are specified in degrees the initial matchup was against the 0.05° gridded products. a: “Non-ship” observations include Bottle, CTD, Drifter, MBT, Moorings, and XBT in these time periods. b: The Analysis is a daily product with a nominal time of midday, therefore a limit of 12 hours will match all *in situ* observations for the specified day.

As with the previous CDR assessment^23^, three categories of validation were performed. “Skin-raw” directly compares the satellite retrieved skin SST with *in situ* data without accounting for the understood geophysical differences between the two^14,31^. This approach was primarily used for internal development and verifying the expected geophysical differences. Secondly, in “skin-skin” validation the *in situ* observations are matched to the satellite time and adjusted to the skin layer temperature. This approach validates the primary satellite retrieval and includes the minimal adjustments to account for geophysical differences at the observation time. Finally, in “depth-depth” comparisons the time and depth adjusted SST at 20 cm and 10:30 h or 22:30 h local time is compared to *in situ* data matched to those standard times. Depth and time adjustments are made using the same skin-effect and diurnal model as used to generate the SST products, so the distinction between skin-skin and depth-depth validation may not be obvious: the key difference is the magnitude of the time correction. In the skin-skin case the time correction is minimal, typically zero-mean, and for *in situ* platforms reporting hourly will be less than 30 minutes. In the depth-depth case the time correction is a function of the satellite overpass time (Fig. 1), reaching a maximum average value of six hours. Thus, depth-depth comparisons validate the full combination of skin SST retrieval and skin-depth adjustment model.

**Fig. 6:**
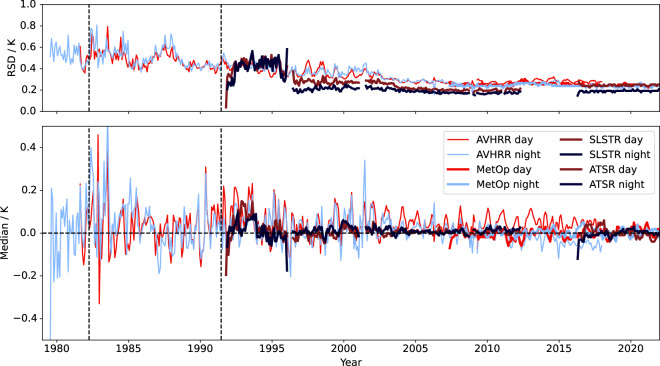
Timeseries of Level 3 validation results. Monthly robust standard deviation (K, top panel) and median discrepancy (K, lower panel) for comparison of SST 0.2 m @ 10:30 local time and reference *in situ*. Vertical dashed lines show time of El Chichón (April 1982) and Mount Pinatubo (June 1991) eruptions.

Comprehensive validation results are available in the PVIR^108^ and a brief overview is presented here. Figure 6 shows the time-series for the depth-depth validation of the infrared sensors (SST CCI ATSR, AVHRR, and SLSTR) using robust statistics. The statistics used are the median, and robust standard deviation (RSD, 1.4826 times the median absolute deviation, a scaling that means the RSD of a normal distribution equals the standard deviation). The relative performance of the various sensors is evident. The dual-view SLSTRs achieve similar accuracy and stability as the ATSR-2 and AATSR sensors. The MetOp AVHRR records are generally more stable around the annual cycle than other AVHRRs. The comparison for AVHRR SSTs in general improves through the timeseries, reflecting improvements in both the satellite instruments and the *in situ* network.

During the first decade there are periods where the CCI AVHRR SST biases fluctuate by a few tenths of kelvin due to problems with the calibration of the early sensors. Coverage is limited prior to September 1981 as we are able to retrieve SSTs from AVHRR/1 instruments only during the night. There is also a short period in early 2001 when the AVHRR SSTs become less stable – this corresponds to a brief period when data was only available from two sensors (AVHRR14 and AVHRR15) both of whose performance was unreliable at this point.

From 2010 to 2018, the CCI AVHRR SSTs from NOAA platforms show a divergence of up to ~0.05 K between the daytime and night-time SSTs. We attribute this to systematic error in the time-of-day adjustment. For these afternoon-orbiting sensors (see Fig. 1), the skin SST observations are retrieved close to the diurnal minimum or maximum in this period, so the required time adjustment incorporated into this depth-depth validation is maximised and most uncertain. The “skin-skin” validation^108^ shows consistency between day and night. (The Analysis SST for this period is more strongly influenced by the Metop AVHRR data because of their coverage at full resolution, and which do not show the same divergence.)

**Fig. 7:**
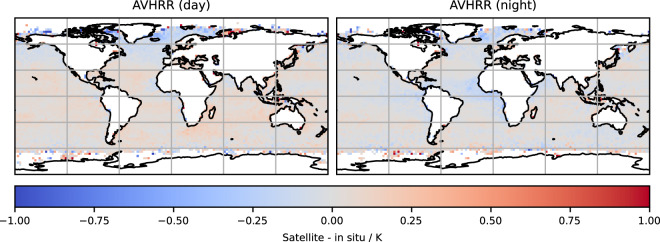
Spatial variation of median CCI AVHRR SST minus reference *in situ* SST (K). Data from all NOAA platforms.

The previous CDR v2.1 was affected by intermittent, localised cold biases in AVHRR SSTs of up to 1 K caused by desert dust^23,49^. These biases have been greatly reduced in version 3 shown in Fig. 7. There are some residual cold biases in the night-time CCI AVHRR SSTs to the west of Africa and in the Arabian Sea of magnitude 0.1 K.

**Fig. 8:**
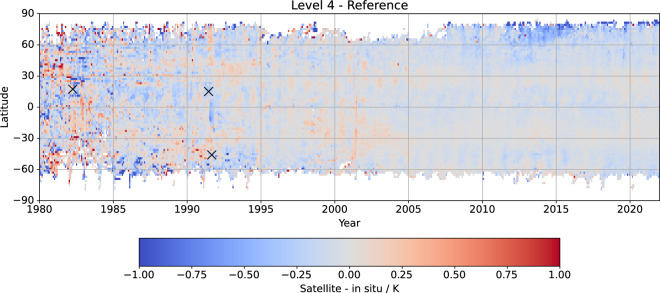
Time/latitude variation of CCI Analysis minus reference *in situ* SST (K) averaged zonally and monthly. Reference *in situ* is drifters-only from 1996, with other sources included prior to this (see Table 12). X symbols mark major volcanic eruptions: El Chichón (April 1982), Mount Pinatubo (June 1991), and Mount Hudson (September 1991).

An overview of the SST CCI Analysis for the complete timeseries is shown in Fig. 8. Differences relative to *in situ* are larger in the early record when the Analysis is based on only one or two AVHRR sensors, although these are reduced compared to the previous CDR. A localised cold bias of a few tenths of kelvin is evident in the tropics immediately following the Mount Pinatubo eruption (1991, marked with a cross). Between 2012 and 2016 there is a cold mean difference in the Arctic of a few tenths of kelvin.

### Validation of uncertainties

**Fig. 9:**
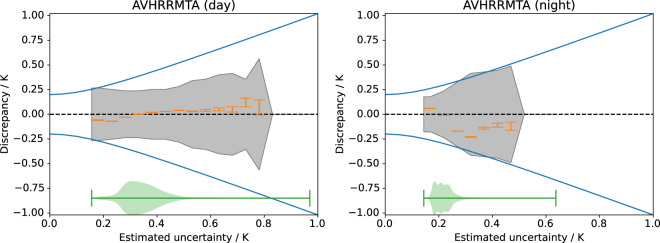
Validation of estimated uncertainty for CCI AVHRR SST from MetOp-A. Plot shows satellite − *in situ* discrepancy against retrieval estimated uncertainty (K). Shaded area shows robust standard deviation (RSD) of difference. Solid blue line shows expected dependence (assuming *in situ* uncertainty of 0.2 K). Orange error bars show median difference for each bin. Green violin plot shows distribution of data.

The uncertainty estimates provided with SSTs are defined as standard uncertainty, i.e., one standard deviation of the estimated error distribution. Uncertainty estimates are validated through comparison against the observed satellite − *in situ* difference distributions as shown in Fig. 9 for MetOp-A AVHRR. These plots compare the observed satellite − *in situ* discrepancy as a function of the estimated uncertainty^33,108^. Given an estimate of the uncertainty in the *in situ* (we assume 0.2 K for drifting buoys for the main distribution, neglecting outliers), the expected spread in the satellite − *in situ* comparison is $\sigma =\sqrt{{\sigma }_{ins}+{\sigma }_{sat}}$. This expected spread is shown as a solid blue line. In this case, the majority of the estimated day-time uncertainties lie between 0.25 and 0.5 K, with extremes up to nearly 1 K. The spread (using RSD) of satellite − *in situ* differences is generally narrower than expected. This indicates that the daytime uncertainties are over-estimated, for reasons we do not yet understand. In the night-time comparison, the estimated uncertainties are mostly less than 0.25 K and in good agreement with the expected satellite − *in situ* spread, meaning the satellite uncertainty estimates are quantitatively credible. The night-time MetOp AVHRR SSTs with larger uncertainties tend to be biased cool relative to *in situ* by about 0.1 to 0.2 K.

Day-time CCI AVHRR SST uncertainties are also found to be over-estimated in general (not shown here). The night-time CCI AVHRR uncertainties and the uncertainties for all other sensors are well estimated.

The spread in uncertainties associated with the CCI analysis SSTs (not shown) is marginally narrower than the expected envelope by 20% to 30%, implying either that the estimated analysis uncertainties are slightly too large, or the assumed *in situ* uncertainty is slightly too large. The CCI analysis SST uncertainties are mostly less than 0.5 K, peaking between 0.25 and 0.3 K, with higher corresponding to areas which were not well observed by the available sensors. These include persistent cloud cover, data gaps in the 1980s, and coastal areas (particularly in the 1980s when only lower resolution AVHRR GAC data is available).

### Climate Assessment

The Climate Assessment Report (CAR)^115^ presents an assessment of trends and variability in the SST CCI products and comparison to other SST products. To assess the multi-annual and decadal behaviour of the new products, comparisons are made to existing long-term (generally coarser resolution) SST data sets used in high profile climate monitoring activities. Differences between the SST CCI products and the comparison datasets are highlighted. The comparison datasets used are: HadSST.4.0.1.0^5^, HadISST1.1^116^, ERSSTv5^117^, COBE-SST2^118^, OI.v2^119^, DailyOIv2.1^120^, CMC v2.0 and v3.0^121^, and gridded drifting buoy and Argo observations taken from the SIRDS. The SST CCI products are also assessed against the precursor SST CCI v2.1 release to determine what progress has been achieved. This process is not validation but does provide important context for potential users to allow them to determine whether the products are credible CDRs and may prove useful. It can also identify features in the SST CCI products that may warrant future investigation or improvement.

Monthly time series of SST anomalies referenced to the SST CCI climatology v3.0 (1991–2020) are calculated and compared for 61 regions of the world’s oceans, including relevant indices, such as for the El Niño Southern Oscillation. Linear trends in these regional series are presented. Maps of decadal average anomalies and their zonal averages are used to identify any large-scale differences between the new products and the comparison data sets.

**Fig. 10:**
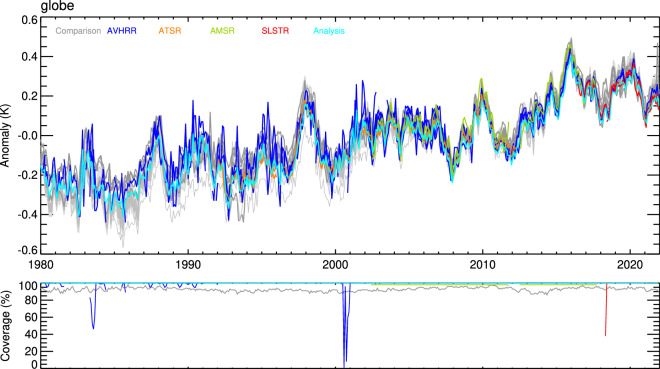
Global average monthly SST anomalies (K, relative to 1991-2020) for the SST CCI products and the comparison data (top panel) and spatial coverage as percent of the global oceans (excluding areas of sea ice) sampled for 5-degree monthly gridded data (lower panel). Anomalies for HadSST.4.0.1.0 comparison data are presented as an ensemble of 200 interchangeable realisations which capture the uncertainty in the bias adjustments applied to the *in situ* observations; the combined uncertainty associated with all measurement and sampling errors is shown as a pale grey envelope enclosing the ensemble. Other comparison data are shown in grey. Anomalies for DailyOIv2.1 comparison data are relatively cool compared to other datasets prior to the mid-2000s and are highlighted in lighter grey. Anomalies for AVHRR-15 from August-October 2000 and SLSTR-B for May 2018 are not shown due to low coverage associated with low data volumes and data beginning on the 30^th^ May respectively. For the comparison data, only coverage for HadSST.4.0.1.0 is shown which approximates the coverage of the combined *in situ* ship and buoy networks.

Some key findings are: (1) The SST CCI products are in good agreement with the comparison data and each other (to order a few tenths kelvin) in terms of resolving global (Fig. 10) and hemispheric climate variability; (2) The SST CCI AVHRR product (and the SST CCI analysis which assimilates it) has been improved versus v2.1, with fewer timeseries spikes, better stability, and better agreement with other SST CCI and comparison datasets, notably in the northern tropical Atlantic and Indian Oceans where the handling of the impact on the retrievals of dust aerosol has been significantly improved; (3) Trends for the SST CCI data and the comparison data are in general in good agreement, including over the full 1980–2021 CDR period, where for most regions the spread of trends is within 0.1 K per decade, comparable to GCOS stability requirements^7^; (4) Regionally the SST CCI AMSR data can be persistently warmer/cooler than the other SST CCI products by a few tenths kelvin and shows relative seasonal anomalies that in higher latitudes can approach several tenths kelvin peak-to-peak magnitude; (5) On decadal timescales a coolness of the SST CCI data (except AMSR) in the mid-high latitudes relative to the comparison data of order a tenth kelvin or more is apparent; (6) The Southern Ocean is a region with relatively greater disagreement amongst the comparison datasets and SST CCI products.

The CAR also presents voluntary reports received from early adopters of the SST CCI v3.0 products, describing their application and what they have discovered from using the data. Overall, the SST CCI v3.0 products represent a mature climate data record for SST and their length of record, stability, resolution and ease of use allow them to be used in a wide range of local and large-scale climate applications.

## Usage Notes

### Known Issues

Coverage is limited in the 1980s – especially prior to the start of AVHRR-7 data in August 1981 (see Fig. 2). Early AVHRRs show intermittent bias fluctuations of a few tenths of kelvin, this is mostly evident in the 1980s, and a brief period at the start of 2001 (see Fig. 6). AVHRR data may be affected by solar contamination of the Earth scene that is not detected in the current version (solar contamination of the calibration system is detected, corrected, and flagged). Smaller coastal features (enclosed bays etc.) cannot be resolved in the AVHRR “GAC” data, these areas will have few or no observations before start of ATSR data in 1991, and the CCI analysis will contain extrapolations from open-sea observations. This will be reflected in the analysis uncertainty, for instance the Seto Inland Sea in the 1980 has a mean uncertainty over 1.5 K.

### Reading the products (quick start)?

All data are stored in netCDF-4 classic format^109^ files following: Climate-Forecast (CF) metadata conventions^110^, GHRSST Data Specifications^10^, and ESA CCI Data Standards^111^. The files make use of “packed” data such that the add_offset and scale_factor attributes must be applied to get values in the correct units – many tools will do this automatically, but users writing low-level code may need to apply the conversion explicitly. The key data variables for the single-sensor Level 2 and 3 products are listed in Table 10, and for the Level 4 SST CCI analysis product in Table 11.

### What software packages can be used to read the data?

Generic Python tools which can be used to read ESA SST CCI data include Iris^122^ and xarray^123^. Two open source toolboxes are available for visualisation and exploration of CCI data: the Climate Analysis Toolbox (Cate) is available from http://climatetoolbox.io/ and the Scientific Toolbox Exploitation Platform (STEP) from http://step.esa.int/main/

### Which product level should I used?

Most users select the Level 4 SST CCI analysis product as it provides an easy-to-use, globally-complete combination of all available sensors, which can be used as a daily average SST at 20 cm depth. However, users should be aware that it only contains the daily average SST, and that the process of interpolation does degrade the feature resolution^124^ which varies spatially and is typically coarser than 15 km (lower than the grid resolution of 0.05°).

Level 3 C data are suitable for users who require convenient single-sensor data on the regular latitude-longitude grid, and who can tolerate spatial data gaps. These files retain all the variables present in lower levels, including both skin and depth adjusted SSTs along with times of observations, though with some spatial averaging to reduce the resolution to 0.05°.

Level 2 P products should be used when the highest possible resolution is required, for example when working on SST features such as fronts and eddies, the disadvantage of this processing level being that SSTs are gappy and located on non-repeating latitude-longitude coordinates.

L3U files are available but are only recommended for specialist users who require gridded data without it being collated to daily files.

### Which type of SST?

There are two different SST estimates in the single-sensor Level 2 and 3 SST CCI products. The first is the primary quantity observed by the sensor: this is the temperature of the skin layer (~10 μm) at time of observations for infrared instruments and the temperature of the sub-skin layer (~1 mm) for microwave instruments^14^. The second is an SST adjusted to represent a depth of 20 cm (comparable to *in situ* drifting buoy measurements) at the 10:30 or 22:30 local time of day (when the diurnal cycle in SST is usually near its daily average). The blended Level 4 product contains a daily SST analysis based on the time-and-depth adjusted Level 2/3 data.

### How should I use the quality and uncertainty information?

Quality 4 and 5 SSTs are recommended for use in climate applications where the absolute accuracy and stability of the SSTs are important. Lower quality levels may be used where maximising coverage is more important than accuracy.

Uncertainty information is provided for the single-sensor products as both a total estimated uncertainty, and broken down into components representing random, correlated, and systematic uncertainties. When using the CCI SSTs at their native resolution users should find the total estimate is sufficient. However, for applications where the data are aggregated to coarser spatio-temporal scales, the errors contributing to the total uncertainty cannot be assumed independent from pixel to pixel. The length scales for the correlated component are not fully quantified, but an appropriate approximation is to treat this component as “systematic” for scales under ~100 km and ~1 day^125^, and “random” at much larger scales.

The Level 4 files contain uncertainty estimates for each SST value. The majority of all data have an analysis uncertainty under 0.5 K, peaking around 0.2-0.3 K. This does increase in coastal and sparsely observed regions and in extreme cases may reach 3 K or more. These higher values indicate that there were no valid SST observations input to the analysis, and the output was effectively extrapolated from more distant (either spatially or temporally) observations.

### How should I refer to the products in publications?

Experience has shown that it is sometimes difficult to infer which datasets have been used in publications, especially as the datasets become available from multiple repositories where they may be presented with different titles. We recommend that the first reference to the dataset in a publication should give the dataset title and/or full dataset name from Table 5 to Table 9 including the version number. After the first usage, the shortened acronym forms are recommended as appropriate. For example, when referring to the products write “using the SST CCI ATSR products” or when referring to the SSTs in a product “frontal features in CCI analysis SSTs”. We strongly encourage restatement of the data version number in captions, legends, slides, or other elements that may circulate independently. Publications should reference this paper and the data citation.

### Will the climate data record be extended in time?

Yes – an extension is available as an interim climate data record (ICDR) which is generated using the same software and systems as the dataset presented in this paper. The version 3.0 SST CCI climate data record covers the period 1980 to end-2021. The Copernicus Climate Change Service (C3S) funded the production of the ICDR for 2022, while the period 2023 and onwards is funded by the UK Earth Observation Climate Information Service (EOCIS, see https://eocis.org) and UK Marine and Climate Advisory Service (UKMCAS). The extension in time is in a delayed mode of up to a month.

## Data Availability

The Multi-sensor Matchup System^114^ is available from https://github.com/bcdev/MMS^127^. SLSTR pre-processing code to regrid Vis/NIR channels to match infrared bands^43^. Code used to validate level 3 and 4 products is available^128^
